# Randomized phase II study of TX followed by XELOX versus the reverse sequence for chemo-naive patients with metastatic gastric cancer

**DOI:** 10.3389/fonc.2022.911160

**Published:** 2022-10-26

**Authors:** Xiao-Yin Zhao, Xin Liu, Wen-Hua Li, Li-Xin Qiu, Ming-Zhu Huang, Chen-Chen Wang, Zhi-Yu Chen, Wen Zhang, Wan-Jing Feng, Wei-Jian Guo, Xiaodong Zhu

**Affiliations:** ^1^ Department of Gastrointestinal Medical Oncology, Shanghai Cancer Center, Fudan University, Shanghai, China; ^2^ Department of Oncology, Shanghai Medical College, Fudan University, Shanghai, China

**Keywords:** Stomach neoplasm (gastric cancer), chemotherapy, docetaxel (DOC), capecitabine, oxaliplatin (L-OHP)

## Abstract

This research found that the clinical outcomes (PFS, ORR, OS) of the non-platinum-based doublet regimen (docetaxel capecitabine combination) were similar to those of the platinum-based (oxaliplatin capecitabine combination) when used as first line therapy for MGC patients.

**Background:**

Docetaxel, platinum and fluorouracil are the three most important drugs in the treatment of MGC. This study was to compare clinical outcomes of the docetaxel capecitabine combination and the oxaliplatin capecitabine combination as first-line therapy in MGC patients.

**Methods:**

In this phase II trial, MGC patients were randomly assigned and treated with either TX (capecitabine 1000 mg/m^2^/twice daily/1-14 days and docetaxel 60/75 mg/m^2^ on the 1st day) (because of toxicity, the dose of docetaxel was reduced to 60 mg/m^2^) or XELOX (capecitabine the same dose with TX and oxaliplatin 130 mg/m^2^ on the 1st day) as first-line therapy. After progression, patients were crossover to the other group as second-line treatment.

**Results:**

Total 134 MGC patients were randomized (69 in TX, 65 in XELOX). There was no significant difference between the PFS of the two groups (TX vs XELOX, 4.6 months vs 5.1 months, p=0.359), and the SFS (9.3 months vs 7.5 months, p=0.705), OS (13.1 months vs 9.6 months, p=0.261), and ORR (46.4% vs 46.2%) were also similar. Among patients with ascites, the TX group had significantly longer PFS and OS than the XELOX group. A total of 85 patients (48 in TX, 37 in XELOX) received second-line treatment, with overall survival of second-line chemotherapy (OS2) of 8.0 m and 5.3 m (p=0.046), respectively. Grade 3 to 4 treatment-related adverse events of first line treatment occurred more in TX group than that in XELOX group(60.6% vs 55.4%).

**Conclusion:**

TX regimen is an alternative choice of first-line treatment for MGC patients. We still need to explore the large number of cohort to confirm this results.

## Background

Gastric cancer is the fifth most common malignancy worldwide (1). The highest incidence is observed in East Asian countries, especially in China and Japan (2, 3). More than 679,000 new gastric cancer diagnoses were recorded in China in 2015 (4). Patients with metastatic gastric cancer (MGC) have a poor prognosis, with a median survival time, if untreated, of 3 to 5 months (5, 6). Unfortunately, although chemotherapy has shown a significant survival benefit, the 2-year OS rate of the MGC is lower than 20% (7). At present, the basic drug for chemotherapy of MGC is fluorouracil, which is included in most combined chemotherapies. The second most important drug is platinum. Conclusively, the most influential guidelines, such as the NCCN and ESMO guidelines, all recommend the combined regimen of fluorouracil and platinum as first-line chemotherapy in MGC (8, 9). The V325 trial showed that adding docetaxel to CF (cisplatin and 5-fluorouracil) significantly improved not only the clinical benefits but also the quality of life (10); therefore, the DCF (docetaxel plus cisplatin and 5-fluorouracil) regimen was once regarded as the preferred regimen. However, the triplet regimen is not very widely used because of the severe toxicity of febrile neutropenia and peripheral neurotoxicity. Although dose reduction of the mDCF regimen achieved an efficacy comparable to that of standard DCF and with fewer toxicities (11–13),But the triplet regimen is still not popular as a first-line therapy due to safety concerns, and therefore, the doublet combination of platinum and fluorouracil remains the most popular regimen.

In several recent phase III trials, docetaxel has shown high activity against gastric cancer and acceptable toxicity as a second-line monotherapy (14, 15). These data confirmed that docetaxel is an important drug in the whole process of palliative treatment of MGC. This finding raises the clinical question of whether the combination of docetaxel and fluorouracil is as effective as the combination of platinum and fluorouracil. However, no randomized controlled trials comparing the efficacy of the taxene-based platinum-free doublet regimen with that of the platinum-based doublet regimen had been performed when we launched this trial. Thus, we designed this study to compare the clinical outcomes of the TX (docetaxel and capcitabine) regimen with those of the XELOX (oxaliplatin and capcitabine) regimen and to identify predictive factors for each regimen.

## Patients and methods

### Patient

The major inclusion criteria were: age 18 years or older; histologically proven gastric or esophagogastric junction adenocarcinoma; measurable and/or assessable metastatic disease according to RECIST 1.1 criteria, or locally recurrent disease associated with one or more measurable lymph nodes; Karnofsky performance status higher than 70; no prior palliative chemotherapy; 6 weeks or longer from prior radiotherapy and 3 weeks or longer from surgery; adequate hepatic, renal, and hematologic function. The major exclusion criteria were concurrent cancer, neuropathy, brain, or leptomeningeal involvement, uncontrolled significant comorbid conditions, and inability to comprehend the purpose of the study or to comply with its requirements. The study was conducted in full accordance with the International Conference on Harmonization Good Clinical Practice guidelines and with the Declaration of Helsinki and was approved by the Ethics Committee of Fudan University Shanghai Cancer, as No. 1204109-14. All patients provided written informed consent before any study procedure.

### Procedure

This is a randomized, single center, open-label, phase II study (The ClinicalTrials.gov ID is NCT01963702) in patients with histologically proven, inoperable, locally advanced or metastatic gastric cancer. Patients were randomly assigned (1:1) to the TX group or to the XELOX group. A computer-generated randomization schedule managed by King Yee Company (Beijing, China) was used. We stratified randomization by number of metastatic sites (≥2 or <2). In the TX group, patients received 75 mg/m^2^ docetaxel (day 1, 1-hour intravenous infusion) plus 1000 mg/m^2^/twice capecitabine (d1-14) once every 3 weeks; in the XELOX group, patients received 130 mg/m^2^ oxaliplatin (day 1, 2-hour intravenous infusion) plus 1000 mg/m^2^/twice capecitabine (d1-14). After the enrollment of 36 patients (19 in the TX group), more than 75% of patients in the TX group developed grade III-IV myelosuppression; hence, the protocol was amended, and the dose of docetaxel was reduced to 60 mg/m^2^ in both TX-XELOX group and XELOX-TX group. Dose modification criteria were predefined. Treatment continued until disease progression, unacceptable toxicity, or consent withdrawal. After 6 courses of treatment, patients whose lesions continue to shrink and who exhibited good tolerability were recommended to receive another 1-2 cycles of treatment, and capecitabine maintenance therapy was recommended for other patients without disease progression. If PD occurred more than 6 months after the end of the first-line treatment, the original regimen could be reintroduced. Upon progression after first-line treatment in each group, patients were recommended to switch to the other regimen (crossover) if possible.

### Evaluation and outcomes

Before random assignment, a complete medical history and physical examination were undertaken, including CBC (complete blood count), blood chemistries, and tumor assessments. Tumor measurements were taken every 6 weeks until progression in both arms and were assessed by the RECIST 1.1 criteria. PFS was measured from the date of randomization to the first radiographically documented progressive disease (PD) or death due to any cause, whichever occurred first. The OS time was measured from the date of randomization to the date of death from any cause. SFS (strategy failure survival) was defined as the time from randomization to progression of the predefined second-line treatment (only TX or XELOX regimens). The medium SFS was calculated in several different circumstances: for patients who died without second-line treatment or who received regimens other than TX or XELOX as second-line therapy, SFS was equal to first line PFS; for patients who remained progression-free until the study cutoff date, SFS was censored and equal to first-line PFS; for patients who crossed over to the predefined regimen, SFS was equal to the sum of first-line PFS and second-line PFS. Patients whose treatments after first-line therapy were not fully collected were excluded when performing the SFS analysis. Toxicities were graded according to the National Cancer Institute of Canada Common Toxicity Criteria, version 3.0. Quality of life was assessed at the same intervals as tumor assessments, and data were collected every 3 months after disease progression using the European Organization for Research and Treatment of Cancer Quality of Life Questionnaire (QLQ) -C30, version 3.

### Statistical analysis

The primary endpoint is the identification potential predictive factors for each regimen, and the secondary endpoints are OS, PFS, ORR, SFS and safety. The Kaplan-Meier method was used to calculate PFS, OS and SFS. Overall response rates were compared using the X^2^ test. PFS and OS were calculated on the predefined full analysis population (all randomly assigned and treated patients). Patients were considered assessable for response if they received two or more chemotherapy cycles and accepted imaging evaluation. Safety analyses included all treated patients and involved the analysis of treatment-emergent adverse events, including events possibly or likely related to study medication and those regardless of causality.

## Results

### Patients characteristics

**Figure 1:**
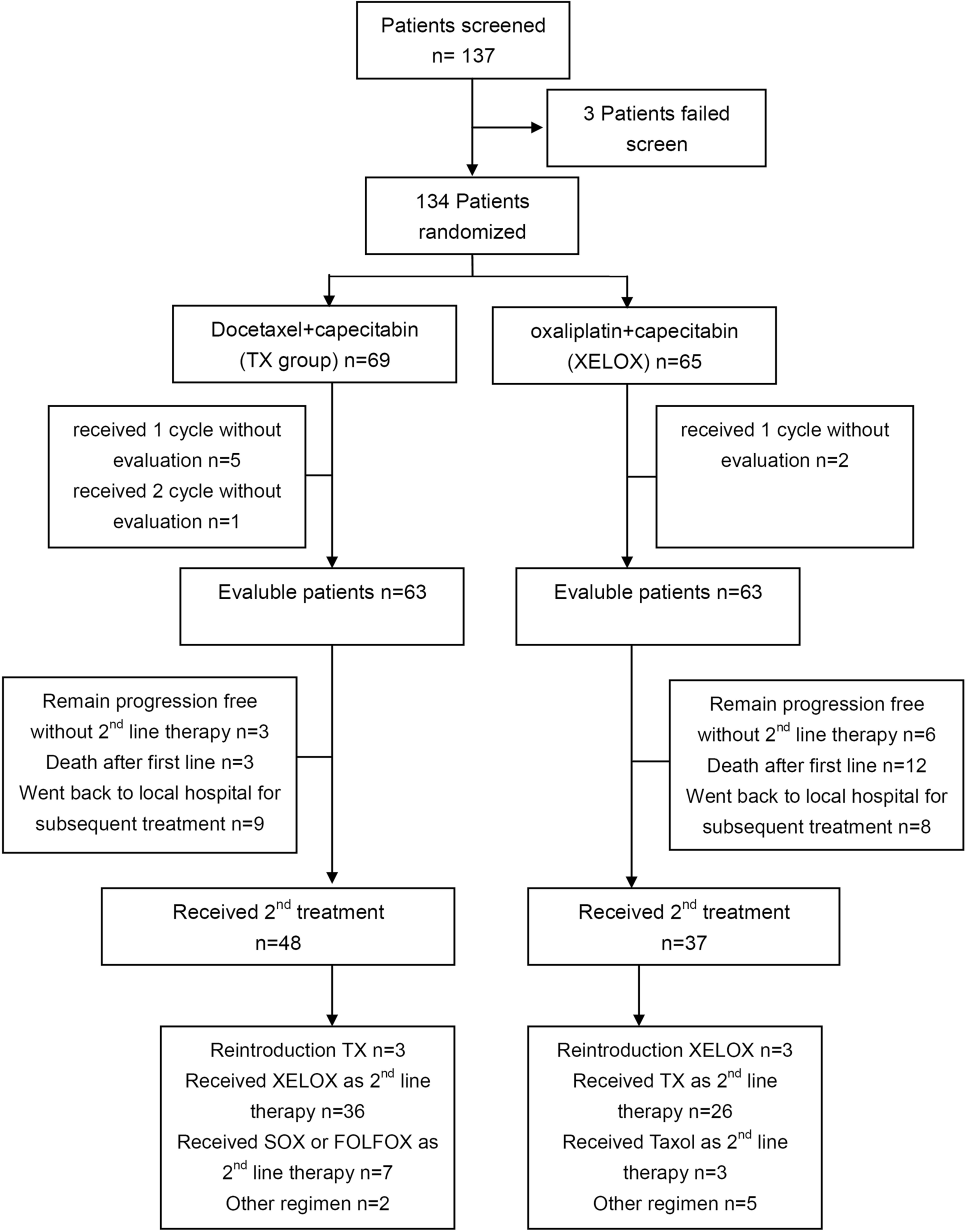
CONSORT diagram.

A total of 137 patients were screened, and 134 patients (TX=69; XELOX=65) were randomly assigned between Aug 2012 and Apr 2015 (**Figure 1**). The 134 patients received the allocated combination, which was considered the intent-to-treat (ITT) population and was analyzed for survival and safety. A total of 126 patients (63 patients in each group) were evaluated and analyzed for response. The majority of patients were males (87 vs 47). More than 80% of the patients had an ECOG score of 0-1. Both treatment groups were well balanced for baseline characteristics (**Table 1**).

**Table 1:** Patient and Cancer Baseline Characteristics.

		Treatment (No. of patients)			
	TX (n=69)		XELOX (n=65)		Total (n=134)	
Characteristic	No.	%	No.	%	No.	%
**Sex**							
**male**	43	62.3	42	64.6	87	64.9
**Age, years**
**Median**	52.1		54.7		53.3	
**Range**	31-71		25-74		25-74	
**<65**	57	82.7	53	81.5	110	82.0
**≥65**	12	17.3	12	18.5	24	18.0
**ECOG**							
**0**	11	15.9	13	20.0	24	17.9
**1**	58	84.1	51	78.5	109	81.3
**2**	0	0	1	1.5	1	0.8
**Primary tumor site**							
**GE junction**	3	4.3	2	3.1	5	3.7
**Fundus**	5	7.2	6	9.2	11	8.3
**Antrum**	50	72.5	48	73.8	98	73.1
**Body**	11	16.0	9	13.9	20	14.9
**Disease status**							
**Locally advanced/recurrent**	3	4.3	1	1.5	4	3.0
**Metastatic**	66	95.7	64	98.5	130	97.0
**No. of organs involved**							
**1**	2	2.9	1	1.5	3	2.2
**2**	18	26.1	19	29.2	37	27.6
**>2**	49	71.0	45	69.3	94	70.2
**Prior therapy**							
**Radiotherapy**	2	2.9	1	1.5	3	2.2
**Surgery**							
**Curative**	7	10.1	6	9.2	13	9.7
**Palliative**	1	1.4	2	3.1	3	2.2
**Adjuvant chemotherapy**	6	8.7	6	9.2	12	8.9

### Chemotherapy

To a total of 134 patients, 359 cycles of TX and 326 cycles of XELOX were administered, with a median of 5.2 cycles with TX (range, 1 to 13) and 5.0 cycles with XELOX (range, 1 to 8). The median duration of therapy was 16 weeks for TX (range, 3 to 65 weeks) and 15 weeks for XELOX (range, 3 to 24 weeks). Dose reductions occurred in 23 patients with TX (33.3%) and 8 patients with XELOX (12.3%). Twice chemotherapy reduction occurred in 6 patients treated with TX (8.7%), and no one in those treated with XELOX. Neutropenia and thrombocytopenia were the most common adverse events leading to cycle delay and dose reduction in TX and XELOX. The most common adverse event leading to dose reduction was neutropenia for TX and thrombocytopenia for XELOX. The main reason for therapy discontinuation was progressive disease in both groups, although this was less frequently observed in patients treated with XELOX than in those treated with TX (89% vs 91%), followed by consent withdrawal (6.1% vs 4.3%), adverse events (1.5% vs 1.4%), death (1.5% vs 2.9%) and other disease (1.5% vs 0%).

### OS and PFS of first-line treatment in ITT patients

**Figure 2:**
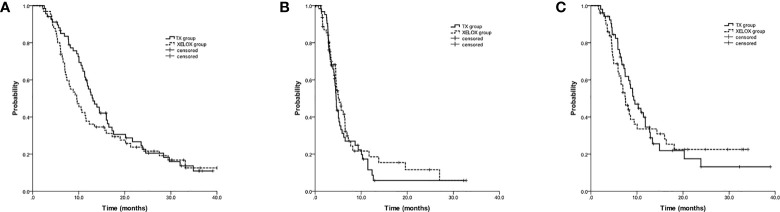
Kaplan-Meier estimates of overall survival **(A)**, progression-free survival **(B)** and strategy failure survival **(C)** among chemotherapy-naive advanced gastric cancer patients treated with docetaxel and capecitabine (TX) or with oxaliplatin and capecitabine (XELOX).

At a median follow-up time of 29.1 months, the median OS was longer with TX versus XELOX (13.1 months; 95%CI, 10.5 to 15.7; vs 9.6 months; 95%CI, 7.1 to 12.1; log-rank p=0.261), albeit with no significant differences (**Figure 2A**). Progression-free survival was similar with TX versus XELOX (4.6 months; 95%CI, 4.2 to 4.9; vs 5.1 months; 95%CI, 4.0 to 6.2; log-rank p=0.359) (**Figure 2B**).

**Figure 3:**
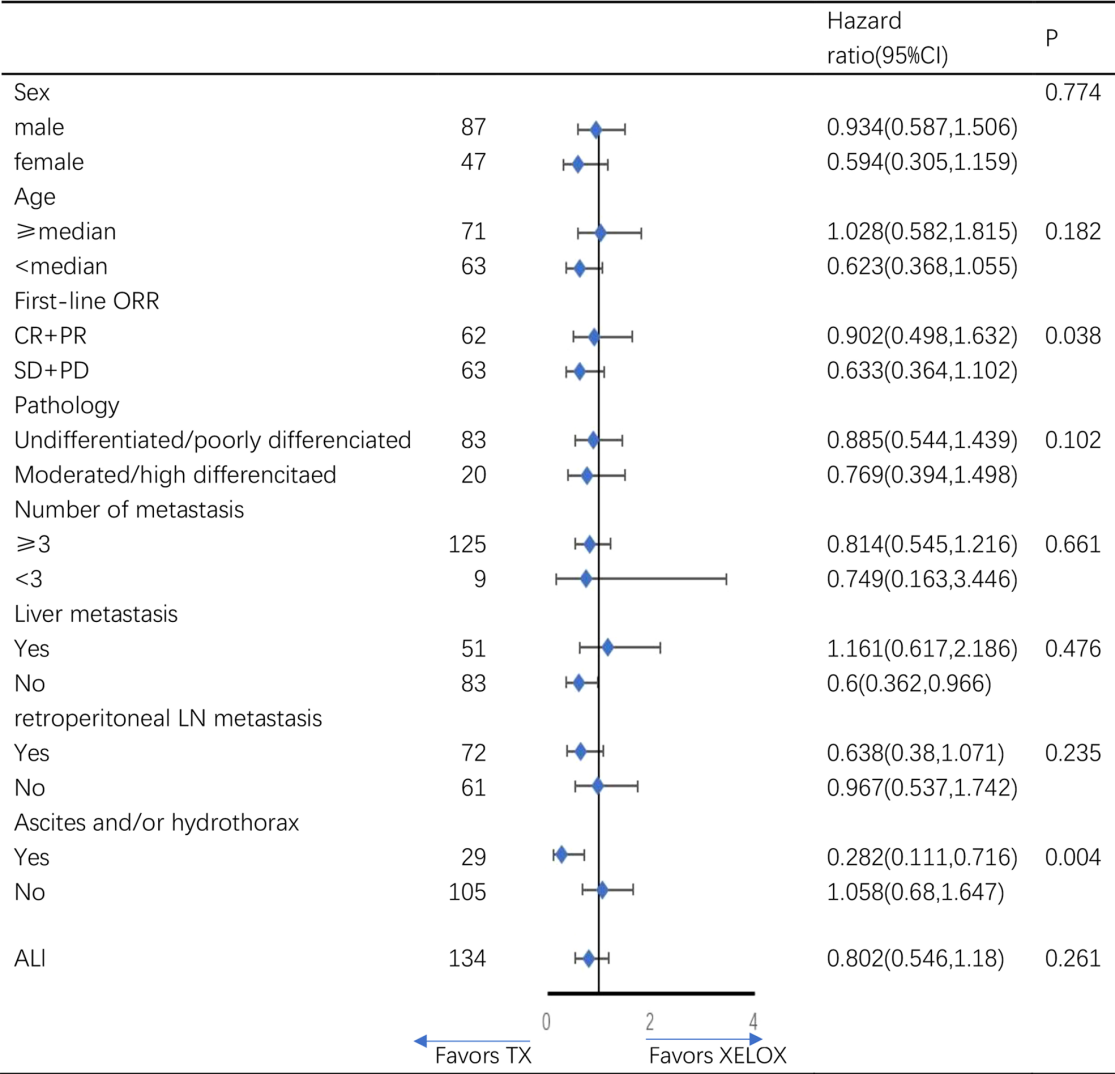
Subgroup analysis of overall survival.

The subgroup analysis for overall survival by baseline characteristics factors showed (**Figure 3**), the TX regimen is more beneficial in patients with ascites and/or hydrothorax(p=0.004), and in patients without liver metastasis(p=0.045). There were no significant difference between TX and XELOX group in subgroup analysis of other baseline characteristics.

### SFS analysis

Among 126 evaluable patients, a total of 104 patients were enrolled in the SFS analysis with sufficient follow-up information. There was no significant difference in SFS time between the TX and XELOX groups (9.3 months; 95%CI, 7.1 to 11.5; vs 7.5 months; 95%CI, 6.0 to 9.0; log-rank p=0.705) (**Figure 2C**).

### Overall response rate

The overall response rate was equal to TX (46.4%) versus XELOX (46.2%) (25%; P = 0.98; **Supplementary Table S1**). No patient had CR. The disease control rate (DCR) was 87.0% in TX and 86.2% in XELOX.

### Second-line treatment

After disease progression, a total of 85 patients received second-line treatment, namely, 48 (69.6%) patients in the TX group and 37 (56.9%) patients in the XELOX group, and 62 patients switched to the other group of treatment (**Table 1**). Three patients in the TX group and six patients in the XELOX group remained progression-free up to the study cutoff date. Three (4.3%) patients in the TX group and 12 (18.5%) patients in the XELOX group died without second-line treatment due to rapid progression or to poor PS status, and the two ratios differed significantly (Fisher exact test p=0.03). In 17 patients (9 patients in the TX group and 8 patients in the XELOX group), the information on follow-up treatment after first-line was not fully collected (The vast majority of them went back to local hospitals for subsequent treatment).

### Survival (OS2) and progression-free survival of second-line therapy (PFS2)

For the 85 patients who received second-line therapy, PFS2 (PFS of second-line chemotherapy) and OS2 (OS of second-line chemotherapy) in the TX group were significantly longer than those in the XELOX group (PFS2, 3.4 months vs 2.4 months, p=0.045; OS2, 8.0 months vs 5.3 months, p=0.046).

**Figure 4:**
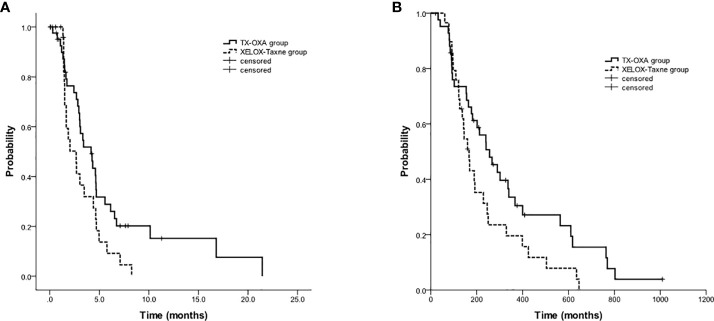
Kaplan-Meier estimates of progression-free survival **(A)** and overall survival **(B)** among 2^nd^-line oxaliplatin-based vs. taxane-based regimens (72 patients).

Among the 48 patients in the TX group, forty-three changed to oxaliplatin-based 2^nd^-line doublet chemotherapy (XELOX, 36 patients; oxaliplatin plus fluorouracil or S-1, 7 patients); among the 37 patients in the XELOX group, twenty-nine patients changed to taxane-based 2^nd^-line doublet chemotherapy (TX, 26 patients; taxane plus fluorouracil or S-1, 3 patients). For the 72 patients who received TX or XELOX as first-line therapy and received oxaliplatin or taxane-based doublet as the 2^nd^-line treatment, PFS2 and OS2 in the TX group were also significantly longer than those in the XELOX group (PFS2, 4.2 months vs 2.7 months, p=0.044; OS2, 8.5 months vs 5.6 months, p=0.05) (**Figure 4**).

### Safety analysis

Grade 3 to 4 treatment-related adverse events occurred in 60.6% (TX) vs 55.4% (XELOX) of patients. Frequent grade 3 to 4 toxicities for TX vs XELOX were neutropenia (60.6% vs 15.4%), febrile granulocyte deficiency (17.4% vs 1.5%), anemia (10.1% vs 10.8%), thrombocytopenia (1.4% vs 15.4%), and all grade peripheral neurotoxicity (11.6% vs 38.5%). The main related, 1^st^ treatment-emergent adverse events are summarized in **Table 2**. In addition, 60 patients’ 2^nd^ treatment-emergent adverse events are fully collected and summarized in **Supplementary Table S2**.

**Table 2:** 1^st^ Line Hematologic and Nonhematologic Toxicities.

	Treatment (No. of patients)							
	TX (n=69)				XELOX (n=65)			
	Grade 3-4		All Grades		Grade 3-4		All Grades	
	No.	%	No.	%	No.	%	No.	%
**Hematology**	42	60.6	63	91.3	19	29.2	56	86.2
**Neutropenia**	42	60.6	61	88.4	10	15.4	36	55.4
**Leukopenia**	28	40.6	56	81.2	5	7.7	30	46.2
**Anemia**	7	10.1	50	72.5	7	10.8	45	69.2
**Thrombocytopenia**	1	1.4	7	10.1	10	15.4	31	47.7
**Febrile neutropenia and/or neutropenic infection**			12	17.4			1	1.5
**Nonhematologic TEAEs**	6	8.7	49	71.0	5	7.7	52	80.0
**Gastrointestinal**	4	5.8	29	42.0	4	6.2	37	56.9
**Stomatitis**	3	4.3	11	15.9	3	4.6	9	13.8
**Diarrhea**	2	2.8	7	10.1	1	1.5	4	6.2
**Nausea**	3	4.3	23	33.3	3	4.6	35	53.8
**Vomiting**	1	1.4	9	13.0	1	1.5	12	18.5
**Anorexia**	0	0	20	29.0	0	0	25	38.5
**Neurosensory**	1	1.4	8	11.6	2	3.0	25	38.5
**Lethargy**	0	0	14	20.3	0	0	10	15.4
**Infection**	1	1.4	7	10.1	1	1.5	4	6.2
**Edema**	1	1.4	3	4.3	0	0	3	4.6
**Hand foot syndrome**	0	0	8	11.6	0	0	7	10.7

## Discussion

The platinum and fluorouracil-based regimen is now widely recommended as first-line chemotherapy in MGC by almost all influential guidelines. Few randomized clinical trials have aimed to determine the role of nonplatinum-based (taxane-based) chemotherapy in MGC. Our study is a randomized clinical trial that aimed to compare clinical outcomes between platinum- (XELOX) and taxane-based (TX) regimens. Our study revealed that the taxane-based TX regimen had similar PFS, OS and ORR to the platinum-based regimen. These results support TX as an alternative to platinum-based regimens and expand the choice of first-line chemotherapy for MGC.

The Japanese START trial (16) is another study that tried to confirm the efficacy of the DS regimen (docetaxol plus S1, an oral fluoropyrimidine, also a nonplatinum-based doublet regimen). The results showed that DS is superior to S1 monotherapy in PFS and OS and comparable to the SP regimen (cisplatin plus S1) of the SPIRITS trial (17). Since the control group of the START trial is S1 monotherapy, the study cannot be directly compared with platinum- and nonplatinum-based doublet regimens.

The trial reported by Lu Z (18) is similar to our study to some extent. These authors tried to prove that the PFS of the paxlitaxel plus capecitabine regimen (PACX) is superior to that of the cisplatin plus capecitabine regimen (XP) in gastric cancer. Although this trial failed to identify the superiority of PACX, the response rate of the PACX group was significantly higher, and the OS was better, albeit nonsignificantly, than those of the XP group.

Our findings, together with these two studies, proved that the clinical outcomes of the nonplatinum-based doublet regimen were similar to those of the platinum-based doublet regimen and supported the assertion that the nonplatinum-based regimen can be an alternative choice for first-line treatment of MGC.

Unlike other studies, our study was also designed to compare the clinical outcomes of two treatment sequences, the TX-XELOX and XELOX-TX sequences. Thus, upon progression after first-line chemotherapy, patients switched to the other group (TX or XELOX) for second-line treatment. There was no significant difference in SFS time between the two treatment sequences. Our study was the first to compare the SFS time between the two treatment strategies. The results indicated that the TX-XELOX sequence is an acceptable strategy in MGC when compared with the XELOX-TX sequence.

According to our results, the TX-XELOX sequence had some advantages over the XELOX-TX sequence in the following aspects.

First, subgroup analysis revealed that in patients with ascites, the PFS and OS of the TX group were significantly longer than those of the XELOX group. This phenomenon implied that taxane might be more effective in patients with ascites. Although this phenomenon requires more data for confirmation, similar results have been published in the Phoenix study (19). The Phoenix study compared intraperitoneal and intravenous paclitaxel plus S-1 (PACS arm) versus S-1 plus cisplatin (SP arm) in MGC patients with peritoneal metastasis. The results showed that in patients with moderate ascites, the PACS arm had significantly longer OS than the SP arm, albeit without significant differences in general. In line with the Phoenix study, our research also suggested that, for patients with ascites, the taxane-based regimen might bring more clinical benefits than the platinum-based regimen.

Second, fewer patients in the TX group died without further treatment for deterioration of physical conditions after the first-line chemotherapy than those in the XELOX group (3 pts vs 12 pts), and more patients had good constitution to receive second-line chemotherapy in the TX group than in the XELOX group (48 pts vs 37 pts).

Third, further analysis showed that patients in the TX group had significantly longer PFS2 and OS2 than those in the XELOX group. We found that the phenomenon occurred not only in the 62 patients who received TX-XELOX or the reverse sequence treatment but also in all 85 patients who received second-line treatment (including 23 patients who received the other regimen as second-line therapy). It may be related to the better tolerance of second-line oxaliplatin than that of second-line docetaxel, which requires large-sample research to confirm.

Fourth, although the frequency of grade 3 to 4 treatment-related adverse events observed in first-line therapy was slightly higher in the TX-XELOX sequence than in the XELOX-TX sequence (60.6% vs 55.4%), adverse events showed an opposite result in the second-line chemotherapy. Since both docetaxel and oxaliplatin will be administered sooner or later throughout the treatment, their toxicity is inevitable. Thus, it is important to arrange the treatment sequence in a more reasonable way. The rationality of the TX-XELOX sequence is that patients usually have better performance status during first-line treatment and are more tolerant to highly toxic therapies, while by second-line treatment, poorer physical conditions are more suitable for less toxic regimen.

Our study also had some limitations. First, research on the primary endpoint, namely, the potential predictive factors, is still underway. The present article only reported the results of the secondary endpoint. Second, we found that the PFS2 and OS2 of the TX-XELOX sequence were better than those of the XELOX-TX sequence. Considering the lack of a validation population and the small sample size, more data are still needed to confirm our results.

In conclusion, compared with the XELOX regimen used as first-line therapy for MGC patients, the TX regimen showed similar PFS, OS and ORR. Compared with the XELOX-TX sequence, the SFS of the TX-XELOX sequence was similar, but more patients received second line treatment, fewer patients died rapidly after progression of first line therapy, and OS2 was more favorable. The TX regimen is an alternative for patients with MGC in first-line therapy.

## Data availability statement

The raw data supporting the conclusions of this article will be made available by the authors, without undue reservation.

## Ethics statement

The studies involving human participants were reviewed and approved by The Ethics Committee of Fudan University Shanghai Cancer, as No. 1204109-14. The patients/participants provided their written informed consent to participate in this study.

## Author contributions

Conception and design: XZ and W-JG. Funding support: XZ. Provision of study materials or patients: X-YZ, XL, W-HL, WZ, W-JG, and XZ. Collection and assembly of data: X-YZ, XL, W-HL, L-XQ, M-ZH, C-CW, and Z-YC. Data analysis and interpretation: X-YZ, XL, and XZ. Manuscript writing: X-YZ, W-JF, and XZ. All authors contributed to the article and approved the submitted version.

## Funding

This study was funded by the National Key Research and Development Program of China (Grant NO. 2017YFC1308900) and the Clinical Research and Cultivation Project of Shanghai Shenkang Hospital Development Center (Grant NO. SHDC12017X01).

## Conflict of interest

The authors declare that the research was conducted in the absence of any commercial or financial relationships that could be construed as a potential conflict of interest.

## Publisher’s note

All claims expressed in this article are solely those of the authors and do not necessarily represent those of their affiliated organizations, or those of the publisher, the editors and the reviewers. Any product that may be evaluated in this article, or claim that may be made by its manufacturer, is not guaranteed or endorsed by the publisher.
