# Supplementary material for: Randomized phase II study of TX followed by XELOX versus the reverse sequence for chemo-naive patients with metastatic gastric cancer
Source: Front Oncol. 2022 Oct 26;12:911160. doi: 10.3389/fonc.2022.911160 (PMC9643736; doi:10.3389/fonc.2022.911160)
Supplement: Supplementary file 1 [file Table_1.docx]

| Table S1. Best Overall Response Rate | | | | |
| --- | --- | --- | --- | --- |
|  | **Treatment (No. of patients)** | | | |
|  | **TX(n=69)** | | **XELOX(n=65)** | |
| Parameter | **No.** | **%** | **No.** | **%** |
| Complete response | **0** | **0** | **0** | **0** |
| Partial response | **32** | **46.4** | **30** | **46.2** |
| Stable disease | **28** | **40.6** | **26** | **40.0** |
| Progressive disease | **3** | **4.3** | **7** | **10.8** |
| Not assessable | **6** | **8.7** | **2** | **3.1** |
| Overall response rate (CR+PR) | **32** | **46.4** | **30** | **46.2** |
| Disease control rate (CR+PR+SD) | **60** | **87.0** | **56** | **86.2** |
