# Supplementary material for: Randomized phase II study of TX followed by XELOX versus the reverse sequence for chemo-naive patients with metastatic gastric cancer
Source: Front Oncol. 2022 Oct 26;12:911160. doi: 10.3389/fonc.2022.911160 (PMC9643736; doi:10.3389/fonc.2022.911160)
Supplement: Supplementary file 2 [file Table_2.docx]

| Table S2. ECOG and 2^nd^ Line Hematologic and Nonhematologic Toxicities | | | | | | | | |
| --- | --- | --- | --- | --- | --- | --- | --- | --- |
|  | **Treatment (No. of patients)** | | | | | | | |
|  | **TX(n=33)** | | | | **XELOX(n=27)** | | | |
| 2^nd^ treatment | **Oxaliplatin-based 2^nd^-line doublet chemotherapy** | | | | **Taxane-based 2^nd^-line doublet chemotherapy** | | | |
| ECOG=0 | 0 | | 0 | | 1 | | 3.7 | |
| ECOG=1 | 33 | | 100 | | 26 | | 96.3 | |
|  | **Grade 3-4** | | **All Grades** | | **Grade 3-4** | | **All Grades** | |
|  | No. | % | No. | % | No. | % | No. | % |
| Neutropenia | 3 | 9.1 | 8 | 24.2 | 7 | 25.9 | 15 | 55.6 |
| Leukopenia | 3 | 9.1 | 7 | 21.2 | 6 | 22.2 | 14 | 51.8 |
| Anemia | 2 | 6.1 | 7 | 21.2 | 5 | 18.5 | 8 | 29.6 |
| Thrombocytopenia | 1 | 3.0 | 3 | 9.1 | 3 | 11.1 | 6 | 22.2 |
| Stomatitis | 0 | 0 | 0 | 0 | 1 | 3.7 | 2 | 7.4 |
| Diarrhea | 0 | 0 | 1 | 3.0 | 0 | 0 | 1 | 3.7 |
| Nausea | 0 | 0 | 1 | 3.0 | 1 | 3.7 | 4 | 14.8 |
| Vomiting | 1 | 3.0 | 1 | 3.0 | 1 | 3.7 | 3 | 11.1 |
| New Neurosensory | 1 | 3.0 | 2 | 6.1 | 3 | 11.1 | 5 | 18.5 |
